# Organizational culture, team climate and diabetes care in small office-based practices

**DOI:** 10.1186/1472-6963-8-180

**Published:** 2008-08-21

**Authors:** Marije Bosch, Rob Dijkstra, Michel Wensing, Trudy van der Weijden, Richard Grol

**Affiliations:** 1 Scientific Institute for Quality of Healthcare, Radboud University Nijmegen Medical Centre, Nijmegen, The Netherlands; 2 Dutch College of General Practitioners, Utrecht, The Netherlands; 3 Scientific Institute for Quality of Healthcare, Department of General Practice, School for Public Health and Primary Care (Caphri), Maastricht University, Maastricht, The Netherlands

## Abstract

**Background:**

Redesigning care has been proposed as a lever for improving chronic illness care. Within primary care, diabetes care is the most widespread example of restructured integrated care. Our goal was to assess to what extent important aspects of restructured care such as multidisciplinary teamwork and different types of organizational culture are associated with high quality diabetes care in small office-based general practices.

**Methods:**

We conducted cross-sectional analyses of data from 83 health care professionals involved in diabetes care from 30 primary care practices in the Netherlands, with a total of 752 diabetes mellitus type II patients participating in an improvement study. We used self-reported measures of team climate (Team Climate Inventory) and organizational culture (Competing Values Framework), and measures of quality of diabetes care and clinical patient characteristics from medical records and self-report. We conducted multivariate analyses of the relationship between culture, climate and HbA1c, total cholesterol, systolic blood pressure and a sum score on process indicators for the quality of diabetes care, adjusting for potential patient- and practice level confounders and practice-level clustering.

**Results:**

A strong group culture was negatively associated to the quality of diabetes care provided to patients (β = -0.04; p = 0.04), whereas a more 'balanced culture' was positively associated to diabetes care quality (β = 5.97; p = 0.03). No associations were found between organizational culture, team climate and clinical patient outcomes.

**Conclusion:**

Although some significant associations were found between high quality diabetes care in general practice and different organizational cultures, relations were rather marginal. Variation in clinical patient outcomes could not be attributed to organizational culture or teamwork. This study therefore contributes to the discussion about the legitimacy of the widespread idea that aspects of redesigning care such as teamwork and culture can contribute to higher quality of care. Future research should preferably combine quantitative and qualitative methods, focus on possible mediating or moderating factors and explore the use of instruments more sensitive to measure such complex constructs in small office-based practices.

## Background

Consistently, studies show that patients with chronic illnesses do not receive optimal treatment [1,2]. Redesigning primary care by separating acute care from planned management of chronic conditions has been proposed to close the quality chasm between current practices and optimal standards [3]. Of all chronic conditions, care for diabetic patients is probably the most manifest and widely spread example of primary care development [4,5]. In the Netherlands, 85% of patients with Diabetes Mellitus type 2 are treated within primary care [6].

The creation of practice teams with a clear division of labour is an important aspect within this context [7]. Nurses and nurse assistants both are generally involved in management of patients with diabetes. Therefore, key elements of teamwork, such as sharing clear goals, division of labour, training and communication [8] are suspected to potentially improve care for these patients [7,9]. Studies showed positive associations between higher levels of teamwork and such outcomes as clinical performance [10], absence of hospital physicians due to sickness [11], job satisfaction [12], and patient outcomes such as satisfaction of patients with their care [12-15]. A related construct that is increasingly described in quality improvement research is organizational culture. This interest is based on the increasing recognition that cultural changes are needed alongside the structural changes to secure gains in quality [16]. Some studies showed that organizational cultures that support teamwork and quality improvement may contribute to achieving high quality care [17-20]. However, it has also been shown that a mix of cultures was associated with higher levels of team effectiveness [21], whereas several other studies failed to find associations between culture and performance [22,23].

In most countries, primary care practices are small, office-based organizations, usually consisting of no more than a handful of people. Although evidence for the possible relevance of teamwork and culture is growing, most evidence for these-intuitively appealing-concepts is based on studies in hospital settings. In this study we therefore investigate whether higher levels of teamwork and specific types of organizational culture are associated to diabetes care in small office-based general practices.

## Methods

### Design and population

The present cross sectional study was embedded in an intervention study, in which 350 practices in three regions in the middle and south of the Netherlands were invited to participate. Forty general practices agreed to participate (response rate 11.4%), and they were paired on stratification criteria and randomly allocated to intervention or control group [6]. A researcher visited intervention practices at the beginning of the intervention period, in February to April 2003, to discuss the current practice procedures for diabetes care with the staff. Situations in which various staff members shared tasks was a special topic of discussion. Then a diabetes passport was introduced, a patient-held booklet with important personal information that can be used to track results, record treatment targets and give (educational) information. The professionals discussed how the passport could best fit in the practice routines and work processes. The researcher summarized the various responsibilities involved in diabetes care and the use of the passport on a desk-top card. In the first three months, patients received their passport. Three months later, a researcher visited the practice to discuss the progress of the project and to see whether the division of tasks was being maintained as planned. After 6 months, all patients completed a short questionnaire on the use of the diabetes passport, after which each practice received benchmarked feedback on the introduction and use of the passports [6]. At post-intervention, in May to July 2004, all practice members in the 40 practices who indicated to be actively involved in medical care for patients with diabetes type II (general practitioners, nurse practitioners, and practice assistants) were invited to complete questionnaires on team climate and organizational culture. Team and culture measures were combined with data of diabetes mellitus type II patients younger than 80. The study was approved by the ethics committee Arnhem-Nijmegen. Written, informed consent was received from all study participants.

### Measures

**Figure 1:**
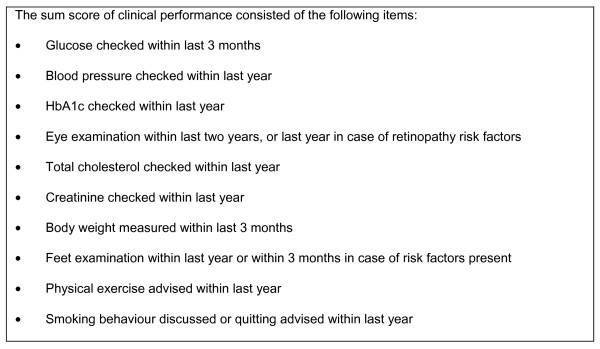
Clinical performance measure: diabetes guideline recommendations.

Clinical outcomes were HbA1c level, systolic blood pressure and total cholesterol levels. A fourth outcome was clinical performance which was measured with a sum score of 10 process indicators of diabetes care quality, based on national guidelines on diabetes care [24] (see Figure 1; measured at the level of the individual patients, Chronbach's alpha 0.86). A patient could be given a score between 0 and 10, because each indicator was scored either done (1) or not done (0). All outcomes were derived by scrutinizing the electronic medical record systems (EMR) by trained research personnel at post-intervention in July 2004.

### Independent factors

To measure organizational culture, we used the 'Competing Values Framework' (CVF) in which respondents were asked to distributed 100 points across four sets of organizational statements according to the description that best fits their own organization in five questions [25]. This approach recognizes that no organization exhibits only one culture or set of values, but that multiple cultures and values coexist simultaneously and compete for attention. The framework distinguishes two dimensions: 'internally oriented' versus 'externally oriented', and 'stability' versus 'flexibility and change', resulting in four ideal types of culture. The *group *culture emphasizes teamwork, cohesiveness, and participation. The *developmental *culture is characterized by the promotion of innovation and risk-taking, and is oriented towards growth. The *rational *culture emphasizes achievement and meeting objectives; people are rewarded to achieve organizational goals and working efficiently. Finally, the *hierarchical *culture emphasizes stability, rules, regulations and coordination. The statements reflect the four culture types. For each question, non blank respondent errors (i.e. the allocation of more or less than 100 points) were corrected by proportionally adjusting the responses to sum up to 100. For each practice, we determined the mean scores on the four types of culture. Internal consistency reliability for the four culture types, using Cronbach's alpha, were 0.64 for group culture, 0.51 for developmental culture, 0.55 for hierarchical culture, and 0.46 for rational culture. In addition, we calculated how well the scores for the different organizational types of culture were in balance, using the Blau index that has been described in previous studies [21,22]. The hypothesis underlying this measure is that it is the relative balance among the four culture types that is associated with team effectiveness. Higher scores on this index indicate a more even distribution of points among the four culture types, so practices that distributed their points in a 25/25/25/25 pattern had the highest score on 'culture balance' (1), whereas practices with more points for one or the other culture type had lower balance scores (< 1).

Teamwork was measured with the 14 item short version of the 'Team Climate Inventory' (TCI) [26,27], answered on 5-point Likert scales. The underlying theory argues that group innovations often result from team activities which are characterized by 1) focusing on clear and realistic objectives in which the team members are committed (vision), 2) interaction between team members in a participative and inter-personally non-threatening climate (participative safety), 3) commitment to high standards of performance and, thus, preparedness for basic questions and appraisal of weaknesses (task orientation), and finally, 4) enacted support for innovation attempts including, e.g. cooperation to develop and apply new ideas (support for innovation). For each scale, mean scores were calculated per individual and then averaged to practice-level scores. Chronbach's alphas were 0.81, 0.79, 0.78, and 0.82 respectively, and correlations (r) ranged from 0.49 to 0.53. We finally combined these to one single score [15]. Overall Chronbach's alpha for the 14 questions was 0.91. Correlations between scales and the overall measure ranged from 0.75 to 0.84.

We translated both the team and culture instruments into Dutch according to guidelines for cross-cultural translation [28]. Analysis of variance tests verified that individual level responses to the culture and team climate instrument could be validly aggregated to the level of the teams for all but one scale. The within-team variability of responses was less than the between-team variability (F values ranging from 2.29 to 3.90 (p < 0.005)). This test was not significant for the hierarchical culture scale (F value 1.3; p = 0.19).

The following-possibly confounding-factors were included: whether the practice had special diabetes consulting hours, and whether it was an intervention or control practice, measured by a checklist that was completed by a member of each practice personnel at the start of the project. Finally, age and gender of the patients were included, derived from mailed patient questionnaires, and the baseline measures of the four outcomes derived from the EMR.

### Analysis

We performed multi level regression analyses (mixed models) with patients (level 1) nested within the practices (level 2). We examined bivariate correlations to check for high correlations (Pearson's correlation and cross tabulations with χ^2 ^test and studied single relationships between the outcomes and all predictors before adding the control variables. Since we were interested in the effect of each of our variables of interest separately (different types of organizational culture and team climate), we used separate models to study one of these variables at a time. Thus, for each outcome, six models were conducted; four different models examined the four organizational cultures, one examined the balance among these culture types, and one examined team climate. Each model controlled for patient age, sex, and the baseline measure on the particular outcome, whether the practice had special diabetes consulting hours, and whether it was an intervention or control practice. All analyses were performed using SPSS version 12.0.1.

## Results

### Practice characteristics

In total, 146 practice members in 40 practices were invited to complete the questionnaires. We obtained team climate and culture data from 92 respondents, 46 general practitioners (response rate 71%), 8 practice nurses (response rate 73%) and 38 practice assistants (response rate 54%) working in 39 practices (overall response rate: 63%). The analysis on organizational culture and team climate was restricted to the practices in which at least two practice members returned the questionnaires. Therefore, we excluded 9 practice members in 10 practices in which this was not the case. The mean number of appointed members per practice was 3.7 (SD 1.0) and did not differ significantly for excluded practices as compared to included practices (3.4, range 2 to 5 and 3.8, range 2 to 6 respectively, p = 0.2). Also, excluded practices were as often single handed practices as included practices (p = 0.3).

Table 1 shows the characteristics of the practices. Single handed practices were underrepresented in our sample as compared to the national mean (40% versus 60%) [29]. Among the four types of culture, group culture by far received most of the points (mean across practices = 51.6), followed by hierarchical (19.7), developmental (16.9) and finally rational culture (11.8). The balance among these values of culture was 0.60 on average. We also explored the data for the dominant culture [17] (the culture scoring highest in each practice; data not shown). In only 3 practices, hierarchical culture received the highest amount of points. All the other practices had a dominant group culture. The overall mean score on team climate was 1.94. Scores on the four scales were 1.84 for vision, 1.83 for participative safety, 1.96 for task orientation and 2.16 for support for innovation; data not shown).

**Table 1:** Characteristics of practices (N = 30)

	%/Mean (SD)
Type of practice (% Single handed)	40%
Special diabetes consulting hours	36.7%
Group culture (0 – 100)	51.6 (13.2)
Developmental culture (0 – 100)	16.9 (7.4)
Hierarchical culture (0 – 100)	19.7 (8.0)
Rational culture (0 – 100)	11.8 (5.6)
Cultural balance (0 – 1)	0.60 (0.10)
Team climate (1 – 5)	1.94 (0.39)

### Patient characteristics

In 40 practices, 2106 patients received questionnaires. Response rates were 68% for the first, and 69% for the second questionnaire, which resulted in data from 993 patients. Since we excluded 10 practices, 241 patients were excluded, leaving 752 patients for this study. Excluded patients did not differ significantly from included patients with respect to age, sex, and our outcomes.

Inspection of Table 2 learns that the mean age of the patients was 63 years, and 48.7% was male. Mean systolic blood pressure was 144.2; mean total cholesterol was 81.5 and mean HbA1c was 7.0. Scores on diabetes care quality differed considerably, and varied from 0 to 9, with a mean score of 5.82.

**Table 2:** Characteristics of patients (N = 752)

	N	%/Mean (SD)
Gender, % male	752	48.7%
Age, years (SD)	752	63.0 (9.7)
Systolic blood pressure (SD)	716	144.2 (19.4)
Total Cholesterol (SD)	716	81.5 (9.6)
HbA1c	696	7.0 (1.2)
Quality of diabetes care (0 – 10)	752	5.82 (2.8)

Table 3 shows that none of the selected clinical patient outcomes (HbA1c, systolic blood pressure and total cholesterol) showed significant associations with team climate or culture. However, we did find significant relations with clinical performance. A higher score on group culture was associated with lower scores on diabetes care quality (p = 0.04) with a coefficient of -0.04. This means that every 10-unit change on the group culture score (e.g. from 20 to 30 points) resulted in a 0.4 lower score on the diabetes care quality indicator. In theory, if a practice would move from the lowest group culture score to the highest (a difference of 55.6 points in this sample), the score on the quality indicator would decrease by 5.6 * 0.4 = 2.24 points. Since the range in the mean scores for the quality indicator was from 0 to 9 points, 2.24 points therefore represents a maximum decrease of 24.9%. In total, 15.6% of the variation in the quality indicator outcome was determined by our model that included group culture of which 2.7% was accounted for by group culture. On the other hand, maintaining a balance between the different culture types was positively associated with quality (β = 5.97, p = 0.03), representing a maximum 27.6% of the nine point practice range in our quality indicator. A 0.1-unit change in the balance score (e.g. from 0.6 to 0.7) resulted in a 0.6 higher score on the quality indicator. Our model including cultural balance explained 16.2% of the variation in the quality indicator, of which 3.5% was explained by cultural balance.

**Table 3:** Associations between team climate, organizational culture and HbA1c, systolic blood pressure, total cholesterol and the aggregated diabetes process quality indicator, measured at patient level (N = 752).

	HbA1c		Systolic blood pressure		Total cholesterol		Clinical performance	
	
	β	95% CI	β	95% CI	β	95% CI	β	95% CI
Group culture	-0.01	-0.02, 0.00	-0.08	-0.25, 0.10	0.00	-0.01, 0.00	-0.04	-0.08, 0.00 *
Developmental culture	0.00	-0.02, 0.01	0.11	-0.16, 0.39	0.01	-0.01, 0.02	0.04	-0.03, 0.11
Hierarchical culture	0.01	0.00, 0.02	0.10	-0.14, 0.34	0.00	-0.01, 0.01	0.03	-0.03, 0.09
Rational culture	0.02	0.00, 0.03	-0.11	-0.44, 0.23	0.00	-0.01, 0.02	0.04	-0.05, 0.12
Cultural balance	1.35	-0.03, 2.72	9.70	-14.53, 33.93	0.65	-0.42, 1.72	5.97	0.66, 11.28 *
Team climate	-0.22	-0.50, 0.05	2.06	-2.53, 6.64	0.09	-0.13, 0.30	-0.57	-1.76, 0.76

* sign < 0.05

## Discussion

Overall, we found that high group culture scores were negatively correlated with adherence to diabetes guidelines in primary care practice (β = -0.04), whereas maintaining a balance among the different types of culture on the other hand was positively correlated to managing diabetes care well (β = 5.97). None of our variables of interest showed associations with our clinical patient outcomes.

### Comparison with other studies

This study confirmed results of recent studies in primary care in the UK, using the CVF, by showing that primary care organizations primarily have group cultures [22,30]. In one of those studies managers of primary care trusts pointed out the possible disadvantages of group cultures, such as a tendency to be 'inward looking'. They expected quality improvement to be hard to achieve unless practices change their culture to one that valued greater collaboration and sharing of expertise, and a willingness to be more flexible in the way that they operated [31]. In our study, high scores on the group culture variable were negatively correlated with indicators for managing care well. This might be explained in light of the suggestion that different culture types are related to those aspects of performance that are valued by that specific dominant culture type [16]. In other words, for example for *changing *routines (in quality improvement projects), a more team-focused and developmental culture type with a focus on flexibility might be helpful in attaining good results, whereas for *performing routine tasks*, such as inspecting feet every 3 months, aspects valued in the more control orientated rational or hierarchical culture types, with a focus on policies, procedures and production might be needed. Therefore, one could also argue that -to reach *and *sustain high quality care for chronic diseases such as diabetes-teams need to find the balance between flexible and control oriented culture types since continuous measuring and improvement, good teamwork, a drive to gain better results, and working according to protocols are equally important. This might be in line with the fact that we found that a high balance between the different types of culture was positively correlated to high quality diabetes care. An earlier study on the role of perceived team effectiveness in improving chronic illness care reasoned that it would be the relative balance among culture values of participation, achievement, openness to innovation and adherence to rules that is most likely to be associated with perceived team effectiveness. Indeed, this study showed an association between a culture balance and team effectiveness, although it was rather marginal [21]. A recent study in primary care hypothesized that a high score on cultural balance would be associated with high levels of team climate, which was not confirmed by the data [22].

Although previous studies suggested the relevance of teamwork in diabetes care [9,13,15], we failed to find significant associations between team climate and our outcomes, as did a recent UK study [22]. Again, the type of outcome might shed some light on this topic, since studies that did find associations often included outcomes such as work satisfaction [12], absence from work due to sickness [11] and satisfaction by patients with their care [12-15]. Interestingly, climate scores were also quite low as compared to other studies [32,33]. This might point to the fact that different practice members involved in diabetes care may not experience their relationships as a 'true' team when it comes to diabetes care [23,34]. The varied nature of clinical problems in primary care practice make team building especially challenging as compared to 'single specialty practices' [8].

Our study failed to find associations between our organizational factors of interest and intermediate clinical patient outcomes. These findings are consistent with recent findings in studying and reviewing the link between safety-factors and risk-adjusted patient outcomes [35,36]. Although the selection of a clinical outcome is recommended, the selection of such a specific variable may just be too narrow to reflect the complexity of modern patient care [37].

### Strengths and limitations of this study

To gain better insight on organizational factors influencing health care quality, it has been suggested that studies should preferably focus on factors on different levels (e.g. organizational as well as team), include patient outcomes and use multi level data analyses to correct for clustering effects [38]. In the current study, we have taken these suggestions into account. However, some limitations need to be addressed.

First, the relative small sample size in our study may have limited the power to find associations. Since general practices are generally small office-based organizations, the number of participants who returned our questionnaire on organizational culture and team climate was relatively low (varying from 2 to 4). Previous studies using the TCI excluded practices if less than 30% of respondents completed questionnaires [12,22]. However, the number of GPs and other care providers per practice seems generally somewhat lower in the Netherlands than in -for instance-UK practices [13,29,39]. In this study, we also excluded the practices in which only one person returned our questionnaire. The low numbers of respondents could impact the validity of our culture and team climate measures. Low Cronbach's alphas for the culture measures for instance, and the low F-value for the aggregation of the scores on the hierarchical culture scale might point to that. In addition, the fact that primary care practices-both in our study and in the UK [22,30] – tend to have predominantly group cultures raises questions about the sensitivity of the CVF in this setting, especially if culture is analyzed as categorical variable. We have taken this point partly into account by using continuous culture variables in the analyses, however, this cannot fully clear away some concerns about the appropriateness of use of this particular instrument in small practices. Although this instrument has some clear advantages over others, such as the fact that it has been used in several other studies in varying settings, and the fact that it measures 'culture typologies' rather than simple variables [16], the factors measured may have a different meaning in different health care settings.

Also, and partly related to our previous point, since climate and culture are considered to be shared attributes, individual measures are aggregated to practice level. Yet, this ignores the fact that different subgroups may have different opinions (for instance general practitioners may experience the culture differently from the practice nurses or assistants) [12,16]. Especially in very small practices (for instance with only one general practitioner and two practice assistants), it is debatable whether the aggregated score is a valid measure of the reality. However, for subgroup analysis researchers would need much bigger samples of respondents, which raises questions about the feasibility of survey based methods in measuring these complicated constructs.

Further, our process measure was assessed by scrutinising the EMR. However, a considerable gap may exist between what the practice members record, and what they actually do in practice. Especially preventive or counselling activities, such as advising physical exercise, have been found to be under recorded [40]. Also, the guideline indicated that smoking behaviour should be discussed with all patients on a yearly basis, even if they are non-smokers. Therefore, we may have underestimated the scores on the quality indicator. However, it is likely that this holds for all practices to the same extent since they all used an EMR. We cannot rule out the possibility though that other confounding factors may have played a role, such as whether or not a physician received feedback or reminders in the EMR, which may have prompted these GPs to perform and register particular preventive activities. At the time of the study, no specific arrangements with insurance companies existed that may have influenced diabetes management. Some practices had a practice nurse who performed tasks related to care for patients with chronic diseases, however, the availability of practice nurses was equal for all regions in the Netherlands. Single handed practices were underrepresented in our study. However, previous research showed no difference in delegation of preventive tasks and treatment of chronic diseases between GPs in single handed practices compared to GPs in group practices [41] so we can assume that our sample is representative for Dutch practices.

Finally, it is important to note that it is not possible to conclude we showed causal linkages between culture and our outcomes, since the results were based on cross sectional data. We therefore do not know whether high scores on group culture lead to poor diabetes management, or -the other way around-practices in which quality of care is managed in a certain way develop certain types of culture, or culture and performance emerge together in a reciprocal and reinforcing manner [16].

## Conclusion

This study contributes to the discussion around the evidence for intuitively appealing features such as culture and teamwork that have been suggested as a lever for health care improvement. We did find some significant associations between culture and high quality diabetes care, but the relations were rather marginal. On the one hand, one could argue that if organizational culture would have only limited influence on many aspects of care during a long period of time, the resultant of that might still add up to a substantial level. On the other hand, feasibility of current measurements of constructs such as climate and culture is still debatable-especially in primary care settings-, given the fact that response rates are low, and scores are aggregated, which causes power reduction and loss of information. Further, we failed to find any associations with our clinical outcomes, which begs the question if and exactly how these constructs can contribute to evidence based care, and -eventually-healthier patients.

Future studies in primary care should preferably combine quantitative and qualitative research methods and use more complex designs to get a better insight into these complex constructs and possibly mediating or moderating factors. Also, it would be worth exploring possible associations between culture and climate and changes in health care quality, as well as the use of other measurement instruments and methods that are more sensitive to -for instance-different subcultures that might exist within organizations, especially in primary care practices where people work in very small teams and deal with a big variety of clinical problems.

## Competing interests

The authors declare that they have no competing interests.

## Authors' contributions

MB, RD, MW, TvdW and RG designed the study. MB performed the data collection and data analyses, and all other authors contributed to interpreting the data. MB wrote the first draft, which was critically revised by RD and then by all others. All authors have read and approved the final manuscript.

## Pre-publication history

The pre-publication history for this paper can be accessed here:


